# Evaluation of Bruton’s Tyrosine Kinase (BTK) inhibition with alternative doses of ibrutinib in subjects with Chronic Lymphocytic Leukemia (CLL)

**DOI:** 10.1007/s00280-025-04753-0

**Published:** 2025-02-28

**Authors:** Aziz Ouerdani, Belén Valenzuela, Nicoline Treijtel, Nahor Haddish-Berhane, Sanjay Desphande, Srimathi Srinivasan, Emma Smith, Juan José Perez Ruixo

**Affiliations:** 1 Department of Clinical Pharmacology & Pharmacometrics, Johnson & Johnson, Beerse, Belgium; 2 Department of Clinical Pharmacology & Pharmacometrics, Johnson & Johnson, Spring House, USA; 3 Department of Clinical Oncology, Johnson & Johnson, Raritan, USA; 4 Department of Oncology Translational Research, Johnson & Johnson, Springhouse, USA; 5 Johnson & Johnson, High Wycombe, UK

**Keywords:** Ibrutinib, Bruton’s tyrosine kinase (BTK), Occupancy, Oncology, Covalent binding model, Mantle cell lymphoma (MCL), Chronic lymphocytic leukemia (CLL)

## Abstract

**Purpose:**

To evaluate alternative ibrutinib dosing regimens that maintain Bruton’s tyrosine kinase (BTK) receptor occupancy over the entire dosing interval for CLL patients using a model-based approach.

**Methods:**

Ibrutinib inhibits B-cell proliferation via irreversible binding of BTK. As IC_50_ is not an appropriate parameter to describe the potency of the inhibition in the presence of a covalent binding inhibitor. A BTK covalent binding model was developed using *k*_*inact*_*/K*_*I*_ as key parameter to account for covalent binding. The ibrutinib-BTK covalent binding model was used to describe the effect of daily doses of 140, 280, 420 and 560 mg on the proportion of subjects with more than 90% BTK inhibition at steady state trough concentrations. Predictive performance of the model was assessed using the available ibrutinib BTK inhibition data following QD dosing. Model-based predictions were used to identify the minimum ibrutinib QD dose that provides more than 90% inhibition in more than 90% of the subjects.

**Results:**

The covalent binding model was able to describe the data and predicted that ibrutinib QD dose reduced from 420 mg to 280 mg or 140 mg may inhibit de novo synthetized BTK efficiently in a CLL population.

**Conclusion:**

Using a model-based approach showed that reducing the ibrutinib dosing regimen to 280 mg QD or even 140 mg in case of adverse events could maintain BTK inhibition over the entire dosing interval.

**Supplementary Information:**

The online version contains supplementary material available at 10.1007/s00280-025-04753-0.

## Introduction

Ibrutinib, the first approved Bruton´s Tyrosine Kinase (BTK) inhibitor, has shown potent clinical activity in a variety of B-cell malignancies and has been globally approved for the treatment of adult patients with Chronic Lymphocytic Leukemia (CLL) and Small Lymphocytic Lymphoma (SLL) [1]. In addition, ibrutinib is approved for Mantle Cell Lymphoma (MCL), Waldenstrom’s Macroglobulinemia (WM) and Chronic Graft- Versus- Host Disease (cGVHD; including pediatric cGVHD). CLL/SLL are B-cell malignancies characterized by the proliferation and accumulation of mature B cells in the blood, bone marrow, lymph nodes and spleen [2]. CLL/SLL is characterized by a spectrum of clinical manifestations, ranging from indolent disease requiring no treatment for decades, to markedly aggressive disease that requires urgent intervention [3, 4].

The B-cell receptor (BCR) signaling pathway plays a key role in the proliferation, differentiation, development, and survival of B-cells [5]. Aberrant BCR activation has been identified as a major pathogenic factor in several B-cell non-Hodgkin lymphoma (B-NHL) subtypes, including CLL/SLL [6]. BTK is a nonreceptor tyrosine kinase which plays a central role in the signal transduction of the BCR and other cell surface receptors, both in normal and malignant B lymphocytes. In the absence of BTK, BCR signaling is insufficient to induce B cell differentiation into mature peripheral B cells [7]. Therefore, BTK inhibition has been recognized as a validated therapeutic target for B-cell malignancies.

Ibrutinib irreversibly inactivates BTK through covalent binding to Cysteine 481 in the ATP-binding pocket. Consequently, reactivation of BTK activity requires de novo protein synthesis [8]. Ibrutinib has low bioavailability due to high first pass metabolism but is readily absorbed with a median time to maximum concentration (t_max_) of 1 to 2 h, distributed extensively to peripheral tissues and rapidly cleared, primarily by CYP3A4 metabolism, with an effective half-life of 4 to 6 h [1]. Therefore, accumulation with repeated daily dosing is minimal.

Data from Study PCYC-04753 in patients with various B-cell malignancies showed that although ibrutinib is rapidly eliminated from the plasma after oral administration, daily (QD) dosing is adequate to sustain > 85% of subjects with BTK occupancy > 90% for 24 h post-dose at dose levels ≥ 2.5 mg/kg [1]. A dose of 420 mg QD was chosen for CLL/SLL to maximize the percentage of patients with > 90% BTK occupancy, thereby accounting for the potential lower exposure for some patients due to PK variability in larger populations. Because ibrutinib binds covalently to the BTK, de novo synthesis rate, degradation rate and BTK baseline level play a key role in the BTK inhibition by ibrutinib. These parameters were evaluated based on BTK inhibition data from CLL patients. No relationship between ibrutinib exposure and the overall response rate (ORR) in subjects with CLL receiving single-agent ibrutinib of 420 mg (Studies PCYC-04753, PCYC-1102, PCYC-1115 and PCYC-1112) was observed, suggesting that systemic ibrutinib exposures obtained at those QD doses are sufficient to provide maximal clinical response.

Therefore, the current recommended ibrutinib dose for the treatment of adult patients with CLL/SLL is 420 mg once daily (QD), which has shown an overall favorable benefit/risk profile across various clinical studies. Although the benefit-risk profile was positive, some patients needed a reduction in dosage due to adverse events (AEs) [9]. While the standard dose of 420 mg is necessary to limit de novo synthesis of BTK protein, adjusting the dosage in response to AEs may result in reduced concentrations of free drug in plasma. Therefore, an adjustment in dose could potentially decrease the occurrence of AEs while still maintaining BTK inhibition. Several small retrospective and real world studies have suggested that reducing the dose of ibrutinib after administration of the standard dose of 420 mg may be sufficient to maximize clinical outcomes, whilst more effectively manage tolerability [10–12].

This analysis investigates whether reduced dosing regimens in response to AEs can sustain BTK occupancy throughout the entire QD dosing interval. The selection of dosing regimens was guided by BTK occupancy data in CLL patients, derived from an ibrutinib-BTK covalent binding model that characterizes the key drivers of the BTK occupancy. Implementing reduced dosing regimens may offer a pathway for better managing treatment-related side effects in patients experiencing tolerability issues with ibrutinib, while preserving optimal efficacy.

## Methods

**Fig. 1:**
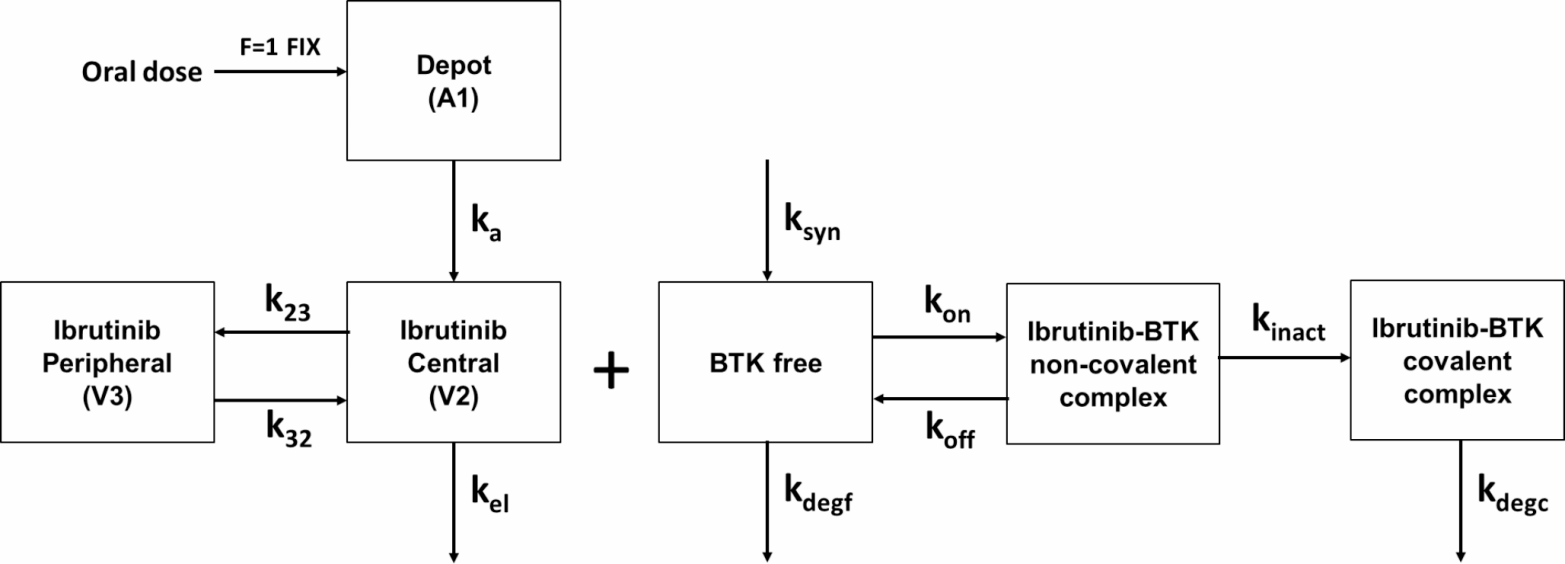
Schematic of the ibrutinib-BTK covalent binding model F: relative bioavailability; k_a_: ibrutinib first-order absorption rate constant from depot to central compartment; V_2_ and V_3_: ibrutinib central and peripheral volumes of distribution, respectively; k_23_ and k_32_: first-order microconstants derived as secondary parameters as Q/V_2_ and Q/V_3_, respectively; k_el_: first-order elimination constant; k_syn_: zero-order production rate constant; k_degf_ = k_degc_: first-order degradation rate constant; k_on_: association rate ibrutinib-BTK binding; k_off_: dissociation rate ibrutinib-BTK binding; k_inact_: covalent binding rate

An ibrutinib-BTK covalent binding model was used to describe the binding of free plasma ibrutinib on its target, free BTK (BTK_f_). This model is composed of three distinct parts: (1) a population pharmacokinetic (PopPK) model describing the time course of ibrutinib plasma concentration and its variability, (2) a model describing the dynamics of the free BTK concentration in absence of ibrutinib, and (3) a model describing the binding of free ibrutinib on free BTK and the inactivation of the reversible ibrutinib-BTK complex. A schematic representation of the model is available in Fig. 1 and each of the three distinct parts of it are described in detail below.

### Ibrutinib population pharmacokinetic model

A previously developed PopPK model [13] was used to simulate total ibrutinib concentrations in plasma. The PK model was an open two-compartment disposition model with sequential zero/first-order absorption after a lag time and first-order elimination from central compartment. The model was parameterized in terms of the apparent clearance (*CL/F*), apparent inter-compartmental clearance (*Q/F*), apparent volume of distribution of the central (*V*_*2*_*/F*), peripheral compartment (*V*_*3*_*/F*), the lag time (*T*_*lag*_), the duration of the zero-order input into the depot compartment (*D*), and the first-order absorption rate constant from depot to central compartment (*k*_*a*_). High-fat meal increased the duration of the zero-order absorption phase from 1.10 to 3.29 h. Relative bioavailability (*F*_*1*_) was fixed to 100% under fed conditions and was estimated to be approximately 67% when the compound was given after an overnight fast. Coadministration of antacids increased *D* by 61%. Allometric relationship was kept on volumes only. Interindividual variability (IIV) on all the PK parameters, except on *k*_*a*_, was characterized with an exponential model. The parameter values are shown in Table 1. The simulated profiles were based on subjects of 80.4 kg (median body weight of the data used to build the popPK model) under fed condition and without coadministration of antacids. In plasma, free ibrutinib concentration was calculated from the total ibrutinib concentration simulated with the model described above and the unbound fraction of 3% [14].

**Table 1:** Summary of the PK and PD parameters used in the simulations

PK Model Parameters [13]			Covalent Binding Model Parameters		
Parameter	Population Mean Estimate	BSV (%CV)	Parameter	Population Mean Estimate	BSV (%CV)
**CL/F (L/h)**	1060	21.9	**BTK**_**f0**_ **(nM)**	1.04^a^	78.9^a^
**V**_**2**_ **/F (L)**	246	153	**BTKf_HL (h)**	60 or 24 or Uniform distribution from 12 to 120	25.0
**Q/F (L/h)**	865	60.7	**k**_**on**_**(1/nM/h)** [25]	1.72 ± 0.60	-
**V**_**3**_ **/F (L)**	9620	47.3	**k**_**off**_ **(1/h)**	0.205^a^	-
**k**_**a**_ **(h** ^**− 1**^ **)**	0.463	-	**k**_**inact**_**(1/h)** [25]	95.76 ± 88.56	-
**Tlag (h)**	0.283	27.8	**FU** [14]	3%	-
**D (h)**	3.29	20.9	**RUV**	27.1	
**F1 for fed patients**	1 FIX	62.8			
**Antiacids on D (factor)**	1.61	-			
**WT_V**	0.641	-			

CL/F: apparent (oral) drug clearance; V_2_/F: apparent central volume of distribution; Q/F: apparent inter-compartmental flow; V_3_/F: apparent peripheral volume of distribution; ka: first-order absorption rate constant; Tlag: temporal delay (lag time) before absorption process is started; D: duration of zero-order input on fed conditions; F1: relative bioavailability after a moderate fast or fed conditions; WT_V: allometric correction for describing the effect of body weight (WT) on volumes implemented as (WT/median body weight)^power^; BSV: between-subject variability; CV%: coefficient of variation (%); BTK_f0_: BTK baseline value; BTKf_HL: BTK half-life; k_on_: ibrutinib-BTK association rate constant; k_off_: ibrutinib-BTK dissociation rate constant; k_inact_: maximum potential rate of inactivation of the covalently bound complex; FU: ibrutinib unbound fraction; RUV: residual unexplained variability expressed as variance

a: data on file

### Free BTK dynamic model

The synthesis rate *k*_*synth*_ and the degradation rate *k*_*deg*_ are the rate constants involved in the zero-order production and the first-order elimination of BTK, respectively. At steady state, the ratio *k*_*synth*_/*k*_*deg*_ provides the baseline level of the free BTK protein (*BTK*_*0*_). The half-life of the free BTK (*t*_*1/2,BTK*_) can be derived from *k*_*deg*_ as shown in Eq. 1.

Eq. 1 $$\:{k}_{deg}=\text{ln}\left(2\right)/{\:t}_{1/2,\:\:BTK}\:$$

*t*_*1/2,BTK*_ measured in vitro ranged from 8 to 12 h [15–17] whereas BTK half-life value estimated from PKPD analyses were around 60 h [18, 19]. BTK half-life in CLL patients remains unclear but the BTK inhibition simulated with the value of 60 h estimated from PKPD analysis in CLL patients [19] seems more consistent with the BTK inhibition seen in Phase 1 and 2 data (PCYC-04753 and PCYC-1102). Therefore, a value of 60 h was used to simulate BTK inhibition of a typical CLL patient whereas a uniform distribution ranging from 12 h to 120 h was used to simulate population of patients with various types of BTK turnover. The lower value of 12 h was chosen based on literature data [15–17] whereas the higher value of 120 h was considered to obtain a twice lower turnover compared to CLL on which BTK half-life was estimated to be 60 h [18, 19].

The parameter values related to BTK dynamics are shown in Table 1. The BTK baseline value (BTK_0_) was 1.04 nM in patients with CLL diagnosis (data on file). The BTK synthesis rate ($\:{k}_{synth}$) was derived from the *BTK*_*0*_ and the *t*_*1/2,BTK*_ values (Eq. 2).

Eq. 2 $$\:{k}_{synth}={BTK}_{0}\times\:\frac{\text{ln}\left(2\right)}{{t}_{1/2,\:\:BTK\:}}$$

### Ibrutinib-BTK covalent binding model

In presence of free ibrutinib, a reversible ibrutinib-BTK complex was generated at an association rate *k*_*on*_. This reversible complex can also dissociate to return free ibrutinib and free BTK entities at a dissociation rate *k*_*off*_. In the context of covalent binding, this reversible complex is mainly inactivated by forming a covalently bound complex characterized by the maximum potential rate of inactivation *k*_*inact*_ [20]. Therefore, the binding constant of the first reversible binding event (*K*_*I*_) is defined as *(k*_*off*_*+ k*_*inact*_*)/k*_*on*_ [21]. Since *k*_*inact*_ > > *k*_*off*_ in case of covalent binding, *k*_*on*_ is approximated to *k*_*inact*_*/K*_*I*_ [22, 23], which is a value available in the literature [25]. Elimination of the covalent ibrutinib-BTK complex was assumed to depend on the longer half-life seen in BTK and so *k*_*degc*_ was equal to *k*_*deg*_. This parameterization suggests that the total BTK concentration is constant over time.

The corresponding differential equations used to describe the time-course of ibrutinib, BTK and ibrutinib-BTK complex based on the full ibrutinib-BTK covalent binding model described above were the following:

Eq. 3 $$\:\frac{\text{d}{A}_{1}}{\text{dt}}=-{k}_{a}\times\:{A}_{1}$$

Eq. 4 $$\:\frac{\text{d}{A}_{2}}{\text{dt}}={k}_{a}\times\:{A}_{1}-{k}_{\text{el}}\times\:{\text{A}}_{2}-{k}_{\text{23}}\times\:{\text{A}}_{2}+{k}_{\text{32}}\times\:{\text{A}}_{3}$$

Eq. 5 $$\:\frac{\text{d}{A}_{3}}{\text{dt}}={k}_{\text{23}}\times\:{\text{A}}_{2}-{k}_{\text{32}}\times\:{\text{A}}_{3}$$

Eq. 6 $$\eqalign{\frac{\text{d}{\text{BTK}}_{f}}{\text{dt}} & ={k}_{syn}-{k}_{on}\times\:{\text{BTK}}_{f}\times\:{C}_{2}+{k}_{off}\cr & \times\:{BTK}_{rc}-{k}_{deg}\times\:{BTK}_{f}}$$

Eq. 7 $$\eqalign{\frac{\text{d}{\text{BTK}}_{rc}}{\text{dt}} & ={k}_{on}\times\:{BTK}_{f}\times\:{C}_{2}-{k}_{off}\cr & \times\:{\text{BTK}}_{rc}-{k}_{inact}\times\:{\text{BTK}}_{rc}}$$

Eq. 8 $$\:\frac{\text{d}{\text{BTK}}_{cc}}{\text{dt}}={k}_{inact}\times\:{\text{BTK}}_{rc}\:-{k}_{deg}\times\:{BTK}_{cc}\:\:\:$$

$$\eqalign{&{with\:k}_{\text{el}}=\frac{CL}{{V}_{2}};{\:\:k}_{23}=\frac{Q}{{V}_{2}}\:;\:\cr & {k}_{32}=\frac{Q}{{V}_{3}}\:and\:{C}_{2}=\:\frac{1000*fu\left(3\%\right)*\frac{{A}_{2}}{{V}_{2}}}{MW\:\left(440.5\right)}.\:}$$

with A1, A2, and A3 being the corresponding ibrutinib amounts in depot, central, and peripheral compartments, respectively; BTK_f_, BTK_rc_, and BTK_cc_ represents the free BTK concentration, the ibrutinib-BTK reversible complex, and the covalently bound ibrutinib-BTK complex, respectively; C_2_ is the free ibrutinib concentration in the central compartment converted in nM using an ibrutinib molecular weight (MW) of 440.5 g/mol [24] and the unbound fraction (fu) of 3% [14]. Other abbreviations were already provided in the body text and in Fig. 1. A summary of the PK and PD parameters used in the simulations are included in Table 1.

### Model-based simulations

Model-based predictions of BTK inhibition were obtained using the ibrutinib-BTK covalent binding model and total daily doses of ibrutinib from 1 mg to 1100 mg administered once daily during 7 days in a virtual population of 10,000 subjects. Uncertainty on *k*_*on*_ and *k*_*inact*_ parameters was included using the 5th, 10th, 20th, 30th, 40th, 50th, 60th, 70th, 80th, 90th and 95th percentiles of 100,000 sampled values generated from a normal distribution of mean 1.72 1/nM/h and 95.76 1/h and standard-deviation of 0.60 1/nM/h and 88.56 1/h, respectively (Table 1) [25]. To tackle the uncertainty of the BTK half-life parameter, BTK half-lives were generated using a uniform distribution ranging from 12 h to 120 h. BTK inhibition was calculated as shown in Eq. 9.

Eq. 9 $$\:{BTK}_{OCC,7;\:i,j}=\:\frac{{BTK}_{rc;\:i,j}+{BTK}_{cc;\:i,j}}{{{BTK}_{f;\:i,j}+BTK}_{rc;\:i,j}+{BTK}_{cc;\:i,j}}\:+{\epsilon}_{i,j}$$

with BTK_OCC,7; i, j_ being the BTK occupancy at trough concentration of last dosing day for the i^th^ virtual subject at the j^th^ dose calculated from the simulated free BTK (BTK_f i, j_) and the reversible (BTK_rc; i,j_) and covalent (BTK_cc; i,j_) complexes. εi_,j_ is a stochastic variable normally distributed with mean zero and variance 27.1 (data on file). The residual unexplained variability was included for model evaluation only. The proportions of patients with more than 90% BTK_OCC,7_ was derived for each dose regimen and summarized. Relationship between BTK occupancy and efficacy is not well established. However, this threshold of 90% is widely used in literature [8, 14].

### Evaluation of the model

To assess the predictiveness of the ibrutinib-BTK covalent binding model, BTK inhibition and proportion of patients with more than 90% BTK occupancy were simulated as described above and compared to the BTK inhibition data from Phase 1/2 study PCYC-04753 and Phase 2 study PCYC-1102.

Table 2 displays the number of BTK inhibition per dose regimen as well as the proportion of patients with more than 90% BTK inhibition. A total of 320 observations were available at pre-dose of days 2, 8, 15 and/or 29 for five tumor type categories and 21 doses ranging from 80 mg to 1400 mg. As the number of BTK inhibition data can be very low in certain dose regimens (*N* < 10), the data were pooled into five dose groups in which the proportions of patients with more than 90% BTK occupancy were calculated: 80 mg to 140 mg, > 140 mg to 300 mg, > 300 mg to 500 mg, > 500 mg to 800 mg and > 800 mg to 1400 mg.

**Table 2:** Number of patients and proportion of patients with more than 90% BTK occupancy per dose

Dose	NObs	% with > 90% BTK Occ
80	8	50
120	12	67
160	7	43
200	12	100
240	7	100
280	4	75
320	4	100
420	119	77
440	7	100
520	4	100
560	49	90
600	8	50
640	13	85
720	4	100
800	4	75
840	40	92
880	4	100
960	1	100
1000	4	75
1060	5	100
1400	4	50
Total	320	82

Nobs: number of observations at predose day 2, 8, 15 and/or 29; Occ: occupancy

### Software

All the simulation analyses were conducted using the R statistical program (version 4.2.0) [26]. mrgsolve [27] and tidyverse [28] R packages were used for simulations, post-processing of the results and data visualization.

## Results

**Fig. 2:**
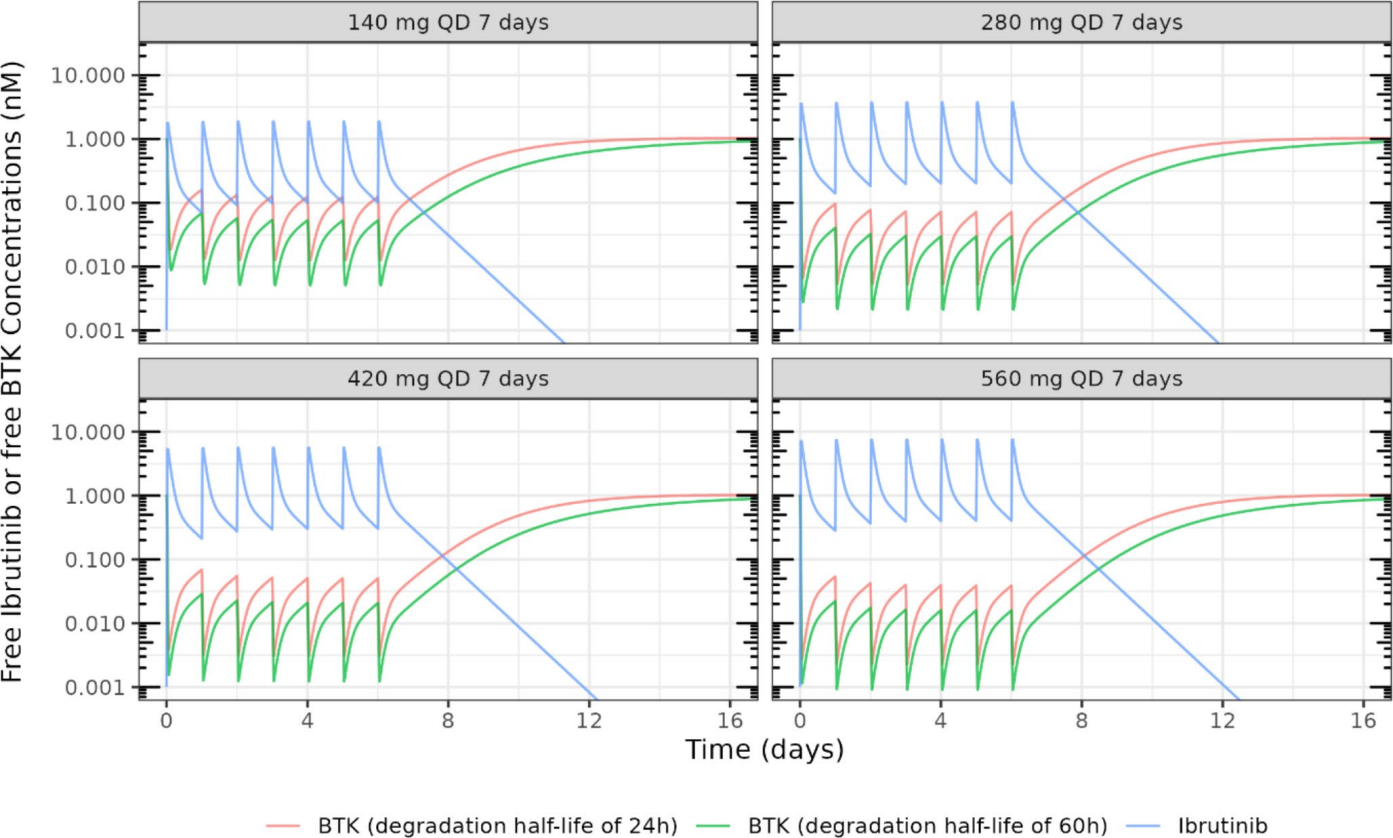
Simulated time course of free plasma concentrations of ibrutinib and free BTK for a typical patient with diagnosis of CLL under different dosing regimens Blue solid lines represent the free ibrutinib plasma concentrations over time. Red and green solid lines represent the free BTK concentrations over time considering a degradation half-life value of 24 or 60 h, respectively

The typical time course of free plasma concentrations of ibrutinib and free BTK for a typical patient with diagnosis of CLL receiving either ibrutinib 140 mg, 280 mg, 420 mg and 560 mg QD for 7 days were simulated using the population PK model, as illustrated in Fig. 2.

As can be seen, ibrutinib plasma concentrations reached steady-state after approximately four days of treatment. The simulated ibrutinib steady-state trough plasma concentration in nM (Day 7) is expected to be 2 to 25 times higher than the corresponding typical free BTK concentration under ibrutinib 140 mg to 560 mg QD (See supplemental material, Table S1). In these four dose regimens, the free BTK concentrations at ibrutinib steady-state trough concentrations are reduced by at least 95% compared to the baseline free BTK concentration. Thereby, in a typical patient with CLL, ibrutinib may almost completely inhibit the baseline plasma BTK and maintain the free BTK concentration at a lower level even at ibrutinib trough concentrations. These results were compared with a scenario where the BTK turnover was faster (BTK elimination half-life of 24 h versus 60 h). Thus, between each ibrutinib administration, free BTK tends to recover towards baseline level more rapidly when a faster BTK turnover is considered. Consequently, free BTK concentrations at ibrutinib trough concentrations are higher and the ratios of free ibrutinib over free BTK are lower (See supplemental material, Table S1). In the four dose regimens simulated for a typical patient with a faster BTK turnover, ibrutinib trough concentrations were able to reduce the baseline BTK level by at least 88%.

**Fig. 3:**
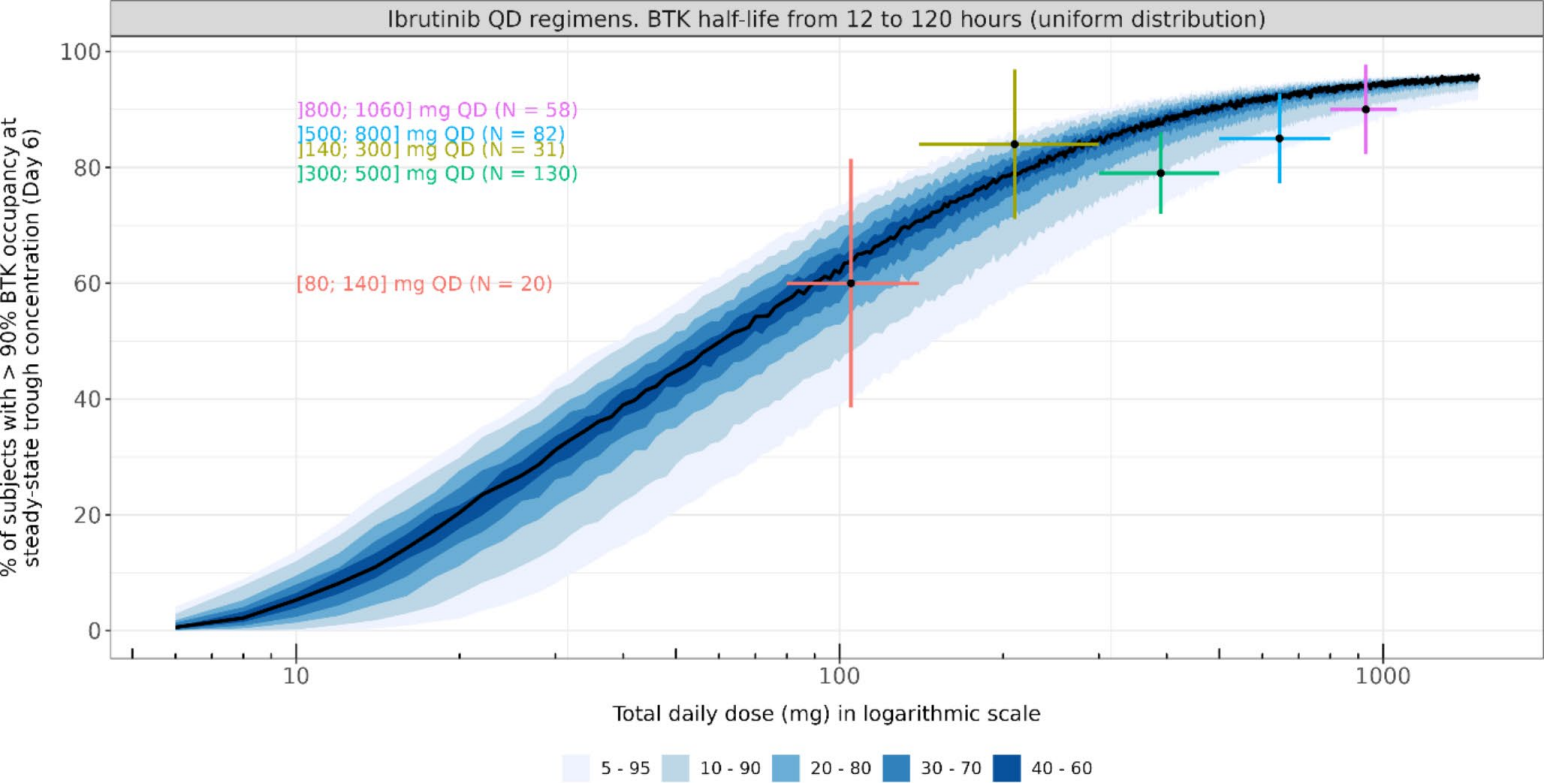
Proportion of subjects with more than 90% BTK inhibition at steady-state trough concentration for ibrutinib QD doses ranging from 1 mg to 1100 mg Black curve and blue areas are the proportion of subjects with more than 90% BTK occupancy simulated with the median and the 5th to 95th percentiles of normally distributed k_on_ and k_inact_ values using means and standard deviations shown in Table 1. Residual unexplained variability was included on the simulated BTK occupancy values to allow comparison with available data. Colored horizontal lines are the observed proportions of subjects with more than 90% BTK occupancy in the different dose groups. N represents the number of observed BTK occupancy data measured at pre-dose of days 2, 8, 15 and/or 29 (Table 2). Vertical colored lines are the 95% confidence interval around the observed proportions

For ibrutinib QD doses ranging from 1 mg to 1100 mg, the expected proportions of subjects with more than 90% BTK inhibition at steady-state trough concentration are displayed in Fig. 3.

For this purpose, 10,000 virtual patients per dose with BTK half-lives generated with a uniform distribution from 12 h to 120 h were simulated. The residual unexplained variability was included for model evaluation only as the simulations aim to be compared with measured BTK occupancy data subject to unexplained error (Eq. 9). As several dose regimens have less than 10 measured BTK occupancy data, observations were pooled into five dosing groups: 80 mg to 140 mg, > 140 mg to 300 mg, > 300 mg to 500 mg, > 500 mg to 800 mg and > 800 mg to 1400 mg (Fig. 3; Table 2). Proportions including residual unexplained variability on BTK occupancy are available in supplementary Table S2. For these 5 dosing groups, observed proportions and their corresponding confidence interval (CI) were included within the predicted 5th to 95th interval of the expected proportions. The covalent-binding model is suitable to describe the relationship between ibrutinib QD dose and the proportions of subjects with more than 90% BTK occupancy at ibrutinib steady-state trough concentration and justify the use of the model to explore alternative QD dosing scenarios that optimize the BTK inhibition.

Intrinsic BTK inhibition (without residual unexplained variability) from 10,000 virtual patients were calculated at the ibrutinib trough concentrations from last dosing day (Day 7, BTK_OCC,7_) for QD ibrutinib at 140 mg, 280 mg, 420 mg, and 560 mg. Median and 95% CI results are shown in Table 3. Median BTK_OCC,7_ was higher than 90% in all four dosing regimens simulated. Except from the 140 mg QD dose, all the simulated ibrutinib dose regimens would provide more than 90% BTK_OCC,7_ in more than 90% of the subjects.

**Table 3:** Simulated median (5th–95th percentiles interval) of BTK occupancy at simulated steady-state trough ibrutinib concentration

Regimen	Median (5th–95th percentiles)	% of patients with ≥ 90% BTK_occ,7_
140 mg QD	94.8 (82.1–97.9)	80.6 (55.0–89.8)
280 mg QD	97.0 (88.9–98.9)	93.3 (75.8–97.2)
420 mg QD	97.9 (91.8–99.2)	96.9 (84.9–99.0)
560 mg QD	98.4 (93.5–99.4)	98.5 (90.3–99.6)

BTK_occ,7_: simulated Bruton’s Tyrosine Kinase occupancy without residual unexplained variability at simulated ibrutinib trough concentration of day 7; QD: once daily

**Fig. 4:**
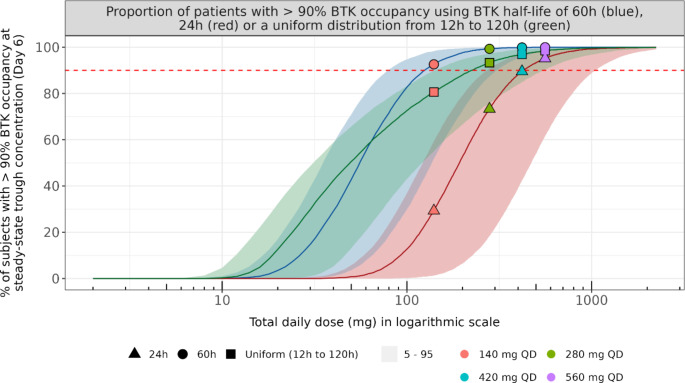
Proportion of subjects with more than 90% BTK inhibition at steady-state trough concentration considering slow, fast, or uniform distribution of BTK turn-over Red, blue and green curves show the simulations on population of patients with a fast turnover (BTK half-life of 24 h with between-subject variability of 25%), a slow turnover (BTK half-life of 60 h with between-subject variability of 25%) and a uniform distribution of BTK turnovers (half-lives ranging from 12 h to 120 h), respectively. Solid curve and colored areas are the proportion of subjects with more than 90% BTK occupancy simulated with the median and the 5th to 95th percentiles of normally distributed k_on_ and k_inact_ values using means and standard deviations shown in Table 1. Colored shapes are the simulated proportions of subjects with more than 90% BTK occupancy at different dose regimens

The proportion of patients with more than 90% BTK_OCC,7_ as a function of ibrutinib total daily dose in the CLL population using three different types of BTK turnover: a fast turnover (BTK half-life of 24 h [17] with between-subject variability of 25%), a slow turnover (BTK half-life of 60 h [19] with between-subject variability of 25%) and a uniform distribution of BTK turnovers (half-lives ranging from 12 h to 120 h) is shown in Fig. 4.

The coefficient of variation of 25% for the between-subject variability of BTK half-life was selected after a sensitivity analysis showing that CV% values up to 75% do not impact the proportion of subjects with more than 90% BTK_OCC,7_. The obtained curves have sigmoid shapes in which the plateaus start to overlay around 1000 mg. The doses leading to 90% of the maximal BTK inhibition (ED_90_) for the 60 h, 24 h and uniform BTK elimination half-lives are 126 mg (95%CI: 80–308), 430 mg (95%CI: 272–1050) and 226 mg (95%CI: 144–552), respectively. The BTK turnover has an important impact on the BTK inhibition and therefore on the proportion of subjects having more than 90% BTK occupancy. A shorter BTK half-life shifts the sigmoid curve to the right side meaning that higher doses are required to keep the same proportion of patients above 90% BTK occupancy. The difference of proportion of subjects with more than 90% BTK_OCC,7_ between the simulations using 60 h and 24 h BTK elimination half-life is exceeding 60% from 70 mg to 150 mg ibrutinib daily doses. This difference does not exceed 15% between the simulations using 60 h and a uniform distribution of half-lives from 12 h to 120 h. This difference drops to 6%, 3% and 1.5% for the 280 mg, 420 mg and 560 mg doses, respectively.

**Fig. 5:**
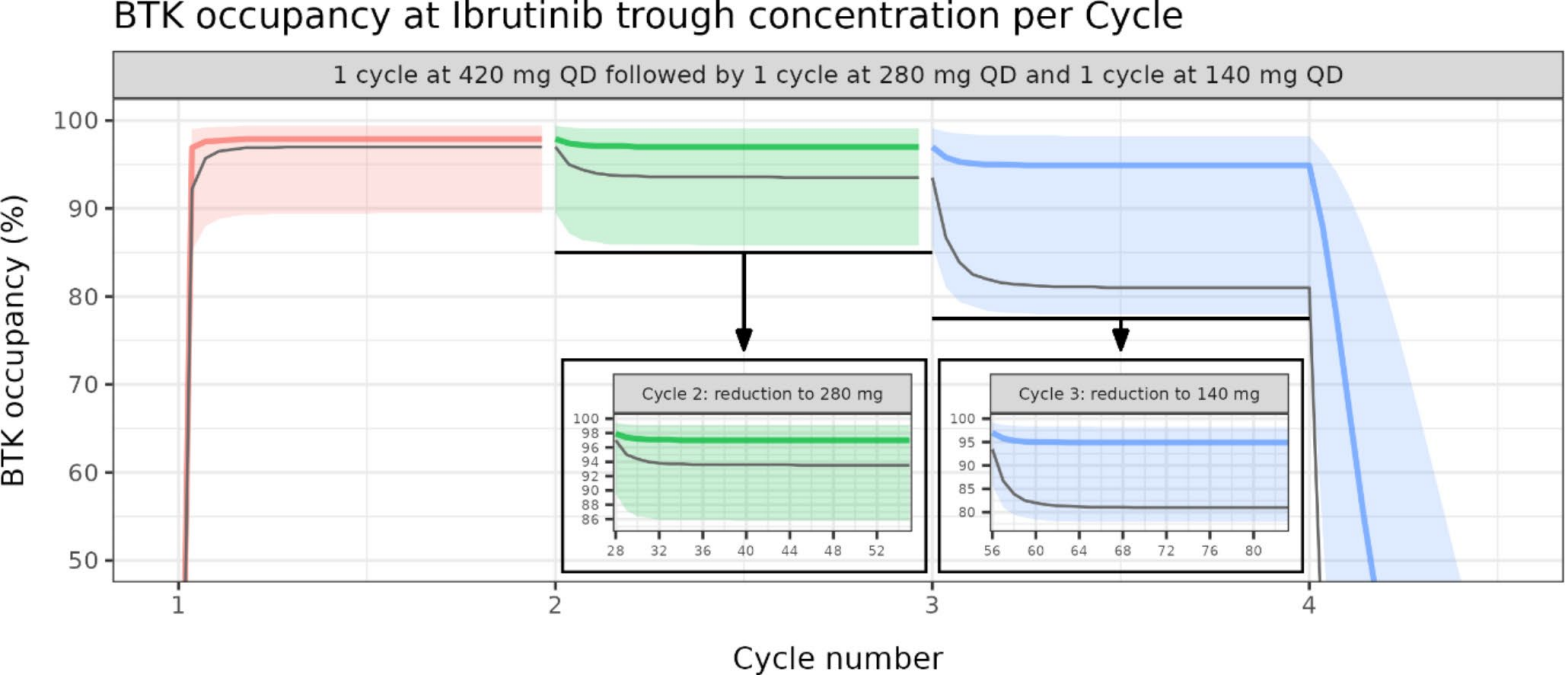
Effect of dose reductions on the BTK occupancy Solid colored lines and shaded areas are the median and the 95% PI of the BTK occupancy at ibrutinib trough concentration derived from a population of 10,000 virtual patients with uniformly distributed BTK elimination half-lives (from 12 h to 120 h). Solid grey line is the proportion of patients with more than 90% BTK occupancy at ibrutinib trough concentration

Finally, the effect on the BTK occupancy when the dose of ibrutinib is reduced from to 420 mg to 280 mg, and to 140 mg (28-days cycle each) was explored (Fig. 5).

A uniform distribution of half-lives from 12 h to 120 h was used to simulate the BTK concentrations. Steady-state is achieved approximately four days into treatment. Transitioning to a 280 mg dose after a cycle at 420 mg results in a slight decrease in the median BTK occupancy from 98 to 97%. The median BTK occupancy drops to 95% when the dose is reduced to 140 mg. The proportion of patients with more than 90% BTK occupancy following dose reduction switches from 97% (steady-state at first cycle of 420 mg QD) to 94% (steady-state at second cycle of 280 mg QD), and to 81% (steady-state at third cycle of 140 mg QD). Therefore, reducing the dose from 420 mg to 280 mg has a limited impact on the BTK inhibition. Despite lower proportions of patients reaching a 90% BTK occupancy, the BTK inhibition remains strong when the dose is reduced to 140 mg. However, the impact on the treatment efficacy, and especially on the progression free survival (PFS) and overall survival (OS), of ibrutinib 280 mg QD and 140 mg QD needs to be evaluated in a clinical trial.

## Discussion

This study describes the potency of BTK inhibition by ibrutinib in CLL patients using covalent binding modeling. A sensitivity analysis was performed to assess the impact on the BTK inhibition of a slow, a fast and a uniform distribution of BTK turnovers using BTK elimination half-lives of 24 h, 60 h, and a uniform distribution from 12 h to 120 h, respectively. In enzyme kinetics with reversible inhibition, the drug concentration that inhibits 50% of the enzyme activity in a biochemical or cellular assay [20], IC_50_, is the key parameter to describe the potency of the inhibition [29] and rank the inhibition potency of different reversible inhibitors. Notably, the IC_50_ is determined by calculating the inhibition of the enzyme at different drug concentrations, and since an equilibrium is obtained between the association and dissociation of the drug-enzyme complex, IC_50_ will not change if different incubation times are used. However, in the context of covalent inhibitors in which a completion of the reaction is attained (rather than equilibrium), IC_50_ becomes dependent on incubation time set for the assay and so different IC_50_ values can be obtained with different conditions of the binding assay. Therefore, IC_50_ is not appropriate to describe the potency of the enzyme inhibition in the presence of a covalent binding inhibitor and, instead, the *k*_*inact*_*/K*_*I*_ should be used as the key parameter describing the efficacy of the irreversible inactivation of the enzyme, in this case BTK [20, 30]. For irreversible inhibitors, this parameter is more adequate than IC_50_ as it permits the interpretation of the selectivity and potency of a drug for its target in a time independent manner [29, 31] and ranking compounds based on irreversible BTK occupancy. The *k*_*inact*_*/K*_*I*_ and *k*_*inact*_ are the key parameters included in the ibrutinib-BTK covalent binding model used in the current manuscript. Thus, published IC_50_ of ibrutinib, zanubrutinib and acalabrutinib were 1.5, 0.5 and 5.1 nM, respectively whereas *k*_*inact*_*/K*_*I*_ were 4.77*10^5^, 2.79*10^5^ and 3.11*10^4^ M^− 1^s^− 1^respectively [25]. Based on *k*_*inact*_*/K*_*I*_ and *k*_*inact*_, ibrutinib is the most potent BTK inhibitor.

Optimal dosing for BTK inhibitors not only depends on the drug potency to inhibit BTK, but also the turnover of the BTK. In that context, Barf et al. suggested that to tackle the higher *de novo* synthetized BTK of aggressive B-cell malignancies with potential faster BTK turnover, higher doses and/or more frequent dosing of acalabrutinib, another covalent BTK inhibitor, might be required to keep an optimal BTK occupancy [32]. Simulation results of ibrutinib and BTK inhibition in CLL population showed that ibrutinib trough concentrations at daily doses from 280 mg were able to reduce the baseline BTK by at least 95% (Fig. 2). Moreover, the impact of faster BTK turnover on BTK occupancy was shown to be maximal between 70 mg and 150 mg ibrutinib daily doses. The difference of proportion of subjects with more than 90% BTK_OCC,7_ between 60 h and 24 h BTK elimination was lower than 30% from 260 mg onwards (Fig. 4). Nevertheless, as the aggressiveness of B-cell malignancies can be highly variable between tumor types or even between patients of a same tumor type such as CLL [33, 34], a population of patients where BTK turnovers uniformly distributed from 12 h to 120 h was simulated and could adequately describe the proportion of patients with more than 90% BTK_OCC,7_ seen in Phase 1/2 study PCYC-04753 and Phase 2 study PCYC-1102 (Fig. 3).

However, for ibrutinib, the QD regimen was supported by exposure-response analyses in patients with CLL receiving single-agent ibrutinib at 420 mg QD, which showed a flat exposure-response relationship for efficacy (ORR) across the observed exposure range, suggesting that systemic exposures obtained at these doses are providing maximal clinical response (data on file). Adapting the dosing regimen of 420 mg QD in CLL patients, may maintain maximal clinical response whilst reducing toxicities. Simulations show that reducing the QD dose of ibrutinib from 420 mg to 280 mg will still provide more than 90% BTK occupancy on at least 96% of the CLL population. In addition, a further reduction to 140 mg could provide more than 90% BTK occupancy on at least 80% of the CLL population (Fig. 5; Table 3). Several retrospective and real-world studies show that dose modification of ibrutinib may resolve AEs without compromising efficacy [10–12]. Building on this evidence, our analysis proposes that reducing the dose of ibrutinib to 280 mg (or even 140 mg) following an initial 28-day cycle at 420 mg could effectively sustain potent inhibition of BTK downstream signaling (Fig. 5). These results are consistent with a pivotal study in which all the patients (*N* = 9) displayed an average BTK occupancy of 95% across the three sequential 28-day cycles of ibrutinib 420 mg, 280 mg and 140 mg once daily [10].

The results of this ibrutinib-BTK covalent binding model add to the emerging body of data which investigates the dose modification of ibrutinib to 280 mg in the clinical setting. In the extended follow-up (up to 8 years) of the pivotal RESONATE-2 study, 90% of the patients had an improvement or resolution of their AEs following a dose reduction [35, 36]. In a real-world analysis of CLL patients receiving either ibrutinib standard (420 mg daily) or reduced doses (either from treatment initiation or within 3 months from starting therapy), overall response rates, PFS, and OS outcomes were comparable between the treatment groups [37]. In another retrospective study of 70 CLL patients treated with ibrutinib, 31.3% of patients required a dose reduction due to AEs or other causes. While there was no significant difference in median PFS and OS between patients who had ibrutinib dose reductions with those on standard ibrutinib dose, the majority of patients (nearly 80%) had their AEs resolved or improved following a dose reduction [12]. In addition, retrospective analyses to determine whether ibrutinib dose modifications correlate with efficacy outcomes in CLL patients treated with ibrutinib outside of a clinical trial context indicate that neither initial ibrutinib dose nor dose modifications during therapy had a negative impact on event-free survival (EFS) and OS outcomes [38].

The above analyses are limited by the small patient sample size and retrospective design. The ongoing phase 2 TAILOR study (NCT05963074) prospectively assesses the impact of both proactive and reactive (per PI) dose reduction of ibrutinib to 280 mg on efficacy and tolerability, for patients receiving ibrutinib continuous and fixed duration ibrutinib and venetoclax regimens.

In conclusion, based on BTK occupancy data generated with the ibrutinib-BTK covalent binding model and based on clinical data, treatment at 420 mg QD has proven to be an optimal efficacious dose that maximizes the proportion of patients with full BTK inhibition. However, doses of 280 mg and 140 mg QD could be appropriate to support side effect management in CLL patients that require dose modifications in response to a TEAE, whilst maintaining a strong inhibition of BTK. The effect of this dose reduction on efficacy endpoints (ORR and PFS) needs to be evaluated in a clinical setting. The ongoing TAILOR study evaluates the impact of the dose reduction strategy at 280 mg on the overall benefit/risk of ibrutinib.

## Electronic supplementary material

Below is the link to the electronic supplementary material.

Supplementary Material 1

## Data Availability

The data sharing policy of Johnson & Johnson is available at https://www.janssen.com/clinical-trials/transparency. As noted on this site, requests for study data access can be submitted through Yale Open Data Access (YODA) Project site at http://yoda.yale.edu.
