# Supplementary material for: Evaluation of Bruton’s Tyrosine Kinase (BTK) inhibition with alternative doses of ibrutinib in subjects with Chronic Lymphocytic Leukemia (CLL)
Source: Cancer Chemother Pharmacol. 2025 Feb 28;95(1):38. doi: 10.1007/s00280-025-04753-0 (PMC11870975; doi:10.1007/s00280-025-04753-0)
Supplement: Supplementary file 1 — Supplementary Material 1 [file 280_2025_4753_MOESM1_ESM.docx]

**Table S1. Free ibrutinib trough concentration and corresponding free BTK concentration in nM at Day 7 per dose regimen.**

| BTK  half-life | Free ibrutinib trough concentration and corresponding free BTK concentration in nM | | | | | | | | | | | |
| --- | --- | --- | --- | --- | --- | --- | --- | --- | --- | --- | --- | --- |
|  | 140 mg QD | | | 280 mg QD | | | 420 mg QD | | | 560 mg QD | | |
|  | Ibr. | BTK | Ratio  Ibr:BTK | Ibr. | BTK | Ratio  Ibr:BTK | Ibr. | BTK | Ratio  Ibr:BTK | Ibr. | BTK | Ratio  Ibr:BTK |
| 60 hours  (reference) | 0.101 | 0.053 | 1.9 | 0.202 | 0.030 | 6.7 | 0.304 | 0.021 | 14.5 | 0.405 | 0.016 | 25.3 |
| 24 hours | 0.101 | 0.124 | 0.8 | 0.202 | 0.072 | 2.8 | 0.304 | 0.050 | 6.1 | 0.405 | 0.039 | 10.4 |

Ibr.: ibrutinib; QD: once daily; BTK: Bruton’s Tyrosine Kinase.

**Table S2. Simulated median (5^th^ – 95^th^ percentiles interval) of BTK occupancy at simulated steady-state trough ibrutinib concentration including the residual unexplained variability**

| Regimen | Median (5^th^ – 95^th^ percentiles) | % of patients with ≥ 90% BTK_occ,7_ |
| --- | --- | --- |
| 140 mg QD | 93.8 (80.0 - 100) | 70.8 (47.8 - 79.8) |
| 280 mg QD | 96.1 (85.0 - 100) | 83.4 (66.2 - 89.3) |
| 420 mg QD | 97.3 (87.4 - 100) | 89.0 (75.2 - 91.8) |
| 560 mg QD | 97.7 (88.2 - 100) | 91.4 (80.4 – 94.0) |

BTKocc: simulated Bruton’s Tyrosine Kinase occupancy with residual unexplained variability at simulated Ibrutinib trough concentration of day 7; QD: once daily.
